# A High-Gain Passive UHF-RFID Tag with Increased Read Range

**DOI:** 10.3390/s16071150

**Published:** 2016-07-22

**Authors:** Simone Zuffanelli, Pau Aguila, Gerard Zamora, Ferran Paredes, Ferran Martin, Jordi Bonache

**Affiliations:** Departament d’Enginyeria Electrònica, Universitat Autònoma de Barcelona (UAB), Campus UAB, Bellaterra, Barcelona 08193, Spain; pau.aguila@uab.cat (P.A.); gerard.zamora@uab.cat (G.Z.); ferran.paredes@uab.cat (F.P.); ferran.martin@uab.cat (F.M.); jordi.bonache@uab.cat (J.B.)

**Keywords:** dipole antennas, impedance matching, radio-frequency identification (RFID)

## Abstract

In this work, a passive ultra-high frequency radio-frequency identification UHF-RFID tag based on a 1.25 wavelengths thin dipole antenna is presented for the first time. The length of the antenna is properly chosen in order to maximize the tag read range, while maintaining a reasonable tag size and radiation pattern. The antenna is matched to the RFID chip by means of a very simple matching network based on a shunt inductance. A tag prototype, based on the Alien Higgs-3 chip, is designed and fabricated. The overall dimensions are 400 mm × 14.6 mm, but the tag width for most of its length is delimited by the wire diameter (0.8 mm). The measured read range exhibits a maximum value of 17.5 m at the 902–928 MHz frequency band. This represents an important improvement over state-of-the-art passive UHF-RFID tags.

## 1. Introduction

The existence of a tradeoff between physical size and performance has always been a key aspect in UHF-RFID tag design. In fact, since the free-space wavelength at the UHF-RFID band (840–960 MHz) is of the order of 30 cm, and tag antennas usually work like resonant dipoles, meandering has always been an extensively used technique in order to avoid excessive tag dimensions, thus allowing the tagging of small items. However, it is well known that dipole meandering decreases the radiation efficiency due to current cancellation [1,2], and thus tag performance in terms of read range is also degraded. Moreover, it is well known that bandwidth is limited by the electrical size of the antenna [3,4] (hence, size reduction goes against worldwide tag functionality). In addition, whereas meandering reduces tag length, it tends to increase tag width, which can be a clear limitation, depending on the aspect ratio of the tagged item.

Whereas for many applications a tradeoff between tag dimensions and performance is necessary, there are some applications where tag performance optimization (i.e., read range) is a due, even at the expense of tag dimensions. Such applications may include, for instance, inventory of boxes in a large warehouse, and pallet tagging, among others. In general, when a high read range is required and the dimensions of the tagged objects are relatively large, oversized (as compared to the usual sizes) tags with optimized read range might be preferred. Moreover, other applications might take advantage of the form factor of a non-meandered wire. For example, the tag could be sewn into the seams of garments, towels, robe and other textile items, or even directly embedded in their fabric by using conductive thread.

In this work, performance optimization of passive UHF-RFID tags without size limitation is studied for the first time, yet keeping the overall size of the tag within reasonable limits for the applications mentioned above. The paper is organized as follows. In Section 2, the tag design principle is discussed. In Section 3, a tag prototype based on a wire antenna and a matching network is presented, along with the electromagnetic simulation results. The discussion is then extended to a case of practical interest in RFID, where the antenna is implemented by means of standard planar technology, providing simulations for the radiation efficiency as a function of the geometry and metal layer conductivity. Finally, the experimental results are reported in Section 4.

## 2. Strategy to Optimize the Read Range

**Figure 1:**
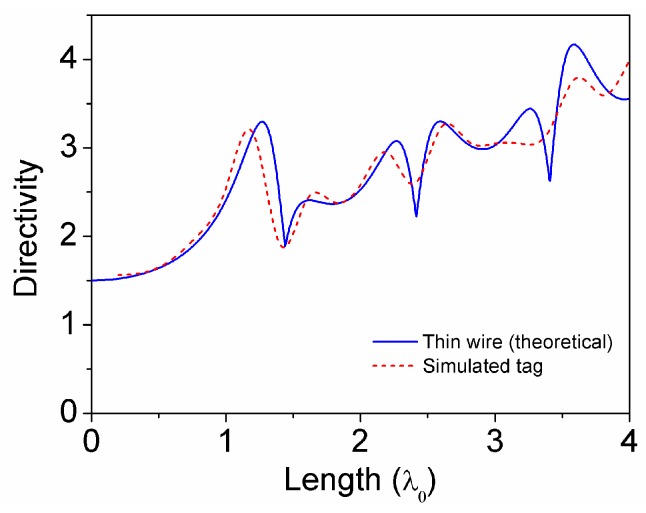
Directivity of a wire antenna as a function of its electrical length.

The field radiated by an infinitely thin wire antenna of a given length *l* oriented along the *z*-axis can be calculated by integrating the radiation of the infinitesimal current element *dI* over the length of the antenna. As reported in many classical analyses of the problem [5,6], it is a good approximation to consider the current over the antenna as a perfect harmonic function of the position, with zeroes at both ends of the wire, thus neglecting the second order effects on the current distribution. The resulting expression for the directivity, as a function of the wire length, can be written as [6]:

(1) $$D_{0}(l)=2\frac{{F(\theta ,l)|}_{MAX}}{\int_{0}^{\pi }F(\theta ,l)\sin(\theta )d\theta }$$

where *F*(*θ*,*l*) is a function proportional to the radiated power density, and can be expressed as:

(2) $$F(\theta ,l)={\left[\frac{\cos\left(\beta \frac{l}{2}\right)-\cos\left(\beta \frac{l}{2}\cos(\theta )\right)}{\sin(\theta )}\right]}^{2}$$

where *θ* is the inclination angle and *β* is the free-space phase constant at the working frequency. A graphical representation of Equation (1) for wire lengths up to four free-space wavelengths is depicted at Figure 1. It can be clearly seen from the graph that the directivity increases monotonically with the antenna length up to approximately 1.25 wavelengths, where a peak value of *D*_0_ = 3.3 (5.2 dBi) results. Above that length, the increase of the side lobes produces a sudden degradation of the directivity, leading to a minimum located at approximately 1.5 wavelengths. A further increase of the antenna length results in periodical directivity peaks and minima, of which levels tend to increase linearly with the antenna length.

Nonetheless, the radiation pattern above the first peak is characterized by a crescent number of lobes, and radiation zeros. Such a kind of radiation pattern is not useful for a general purpose RFID tag, since it produces undesired blind spots in the tag read range. From Figure 1 it is also clear that, above the first directivity peak, the following major peak occurs at 3.6 wavelengths, which implies tag lengths exceeding one meter.

**Figure 2:**
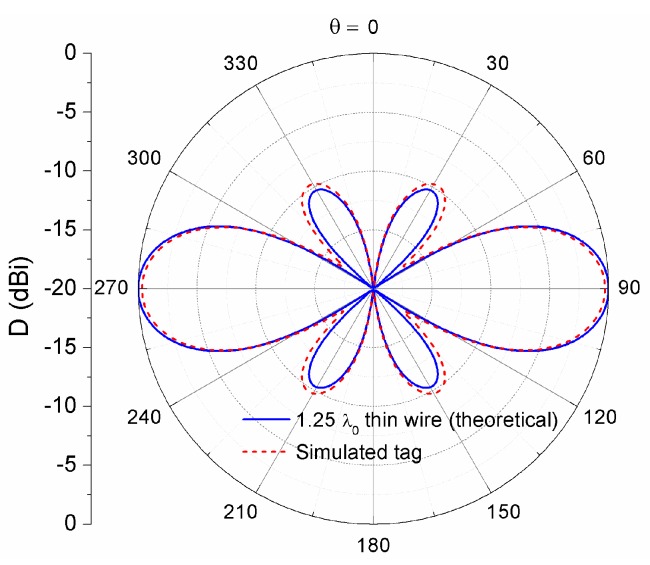
Directive gain in the *e*-plane, normalized to a directivity value of *D*_0_ = 5.2 dBi.

For the above reasons, we found reasonable to design the tag to work at 1.25 wavelengths, corresponding to an overall length of *l* = 410 mm at the frequency *f*_0_ = 915 MHz. The theoretical power radiation pattern, obtained from Equation (1) and normalized to 5.2 dBi, is depicted in Figure 2. It is composed of a main lobe with a maximum at *θ* = 90°, and two side lobes of magnitude −10 dB. The half-power beamwidth is 34°, and the null-to-null beamwidth is 74°. Since the read range of an RFID tag is proportional to the square root of the receiving antenna gain (see Equation (3)), it is interesting to quantify the quarter-power beamwidth, thus corresponding to the half-read-range beamwidth, whose value in our case is 46°. It is worth mentioning that, for a non-zero radius wire, the effective antenna length can differ slightly from the theoretical value of 1.25 wavelengths.

Let us now quantify the maximum theoretical read range which can be obtained with this kind of tag. This can be accomplished by using the very well-known read range formula [7]:

(3) $$r=\frac{\lambda }{4\pi }\sqrt{\frac{EIRP\cdot G_{r}\cdot \tau }{P_{th}}}$$

where *EIRP* is the equivalent isotropic radiated power, *λ* is the free-space wavelength at the working frequency, *G_r_* is the gain of the tag antenna, *τ* = (1 − |*s*|^2^) is the power transmission coefficient and *P*_th_ is the chip sensitivity. By neglecting ohmic and mismatch losses, and considering an *EIRP* level of 4 W and a sensitivity of −17 dBm (e.g., Alien Higgs-3 chip, SOT-323), the maximum predicted read range is *r*_0_ = 21 m. Such value, qualitative since losses have been neglected, is very promising since it doubles the maximum read range of general-purpose commercial UHF-RFID tags (e.g., the Alien ALN9640 “Squiggle” tag [8]), which is of the order of 10–11 m.

It is worth mentioning that, according to the previous analysis, the read range is increased by making the tag more directive. Hence, due to its reduced beamwidth, the tag performance is more dependent on its orientation with respect to the reader antenna. Although this is generally an undesired behavior in general purpose UHF-RFID tags (where the tag orientation is not known a priori), it is not necessarily an issue for many applications requiring read range optimization (e.g., when the tag orientation is known, as in many scenarios of pallet and box inventory). In other applications, like passive RFID sensing, the orientation dependence can even be an advantage, so that a long distance passive orientation sensor could be built based on the proposed antenna. In this latter case, however, ambiguous result may arise due to the presence of the side lobes. Nevertheless, the spurious read range is only 30% (on the basis of Equation (3), and considering a simulated side lobe level of −10 dB) of the peak read range, so that ambiguity could be eliminated by properly distancing the objects from the reader.

## 3. Tag Design and Simulation

### 3.1. Copper Wire Antenna

In this section we present the simulations of a UHF-RFID tag based on a 1.25 wavelength long wire antenna and the Alien Higgs-3 RFID chip (SOT-323 packaged), designed to work in the 902–928 MHz band. For the electromagnetic simulations the commercial software CST Microwave Studio was used.

**Figure 3:**
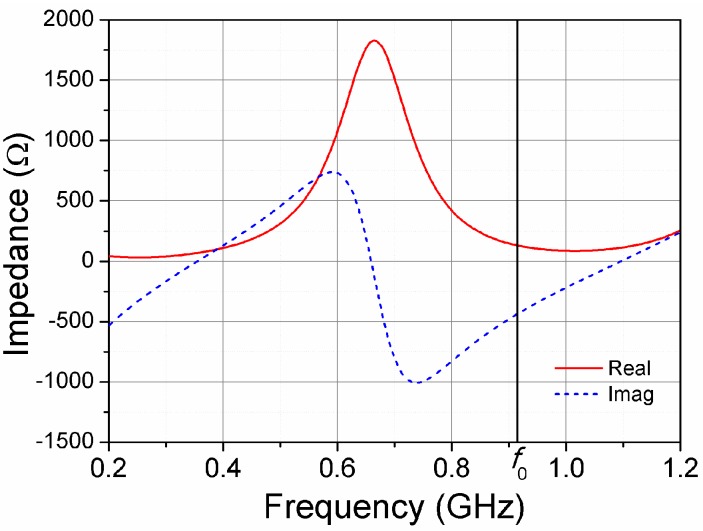
Simulated input impedance of the wire antenna.

The first simulation involved a copper wire with circular cross section of diameter a = 0.8 mm. A 4 mm gap was opened at the center of the wire in order to connect the input port. The wire directivity was simulated as a function of its electrical length (see Figure 1 for a comparison with the theoretical thin wire case), in order to determine the optimal length for the given wire diameter value a. It was found that the length *l* = 400 mm (1.22 *λ*) provided the maximum directivity D = 4.9 dBi, which is very close to the theoretical value (5.2 dBi). The next step was to quantify the input impedance at the working frequency *f*_0_ = 915 MHz (the center of the 902–928 MHz band), in order to design the impedance matching network between the chip and the antenna (see Figure 3). The antenna presents a resistance R_A_ = 130 Ω and a capacitive reactance χ_A_ = −440 Ω at the working frequency *f*_0_, while the chip input impedance (which can be modelled by a shunt RC circuit with values R_c_ = 1500 Ω and C_c_ = 0.9 pF) is Z_c_ = 25 − j190 Ω at *f*_0_. It can be demonstrated, e.g., by using the resistance scaling technique described in Reference [9], that a shunt inductance with value L_p_ = 24 nH transforms the chip impedance Z_c_ into the complex conjugate of the antenna impedance Z_A_ to a very good approximation, thus allowing to obtain a very good impedance (conjugate) matching without the need of a series element. Thus, with the inductance in parallel with the chip, conjugate matching is achieved.

**Figure 4:**
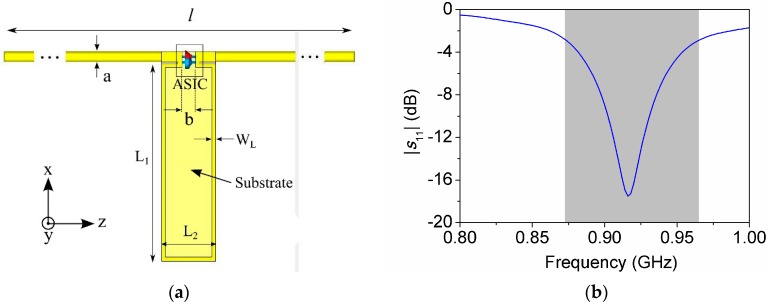
(**a**) Final tag layout; (**b**) Simulated power reflection coefficient (half-power bandwidth in grey).

A second, and last, simulation comprises the wire antenna, the matching inductive element and the chip port exciting the system. The inductive element was implemented on a FR-4 substrate (ε_r_ = 4.3, tan*δ* = 0.025) with thickness h = 1.5 mm, which has also the function of carrying the soldering pads for the chip and the antenna. The final layout of the tag is shown in Figure 4a, and the values for the geometric parameters are L_1_ = 14.6 mm, L_2_ = 4 mm, W_L_ = 0.2 mm, a = 0.8 mm, b = 1 mm, l = 400 mm. The simulated power reflection coefficient between the chip and the antenna, depicted at Figure 4b, exhibits good impedance matching at the central frequency, and a half-power bandwidth of BW = 90 MHz.

Let us now examine the radiation properties of the tag, in order to predict the maximum read range. As can be seen in Figure 2, the simulated radiation pattern (normalized to D_0_ = 5.2 dBi) of the tag is in good agreement with the theoretical pattern of a 1.25 wavelength long wire antenna, as calculated by using Equation (1). The simulated directivity is 4.9 dBi, and the half power beamwidth is 33°. The effect of ohmic losses introduced by the conductors is taken into account in the simulation, resulting in a radiation efficiency of *η*_rad_ = 92%, which leads to a realized gain of G_r_·*τ* = 4.4 dB. Based on these results, the tag read range can be predicted by using Equation (3), providing r = 19.4 m.

### 3.2. Planar Technology

It is interesting to explore the possibility of designing this kind of tag in planar technology, by means of standard fabrication techniques (e.g., inlay, screen printing, etc.). In this case, the conductor has a rectangular section of width w and thickness *t*. The main concern about this implementation is the radiation efficiency, which is a function of the metal conductivity and its thickness. It is important to highlight that this kind of tag is focused on read range optimization, at the expense of its length, and this is possible only if the radiation efficiency remains sufficiently high (*η*_rad_ = 90% can be considered as a lower bound).

**Figure 5:**
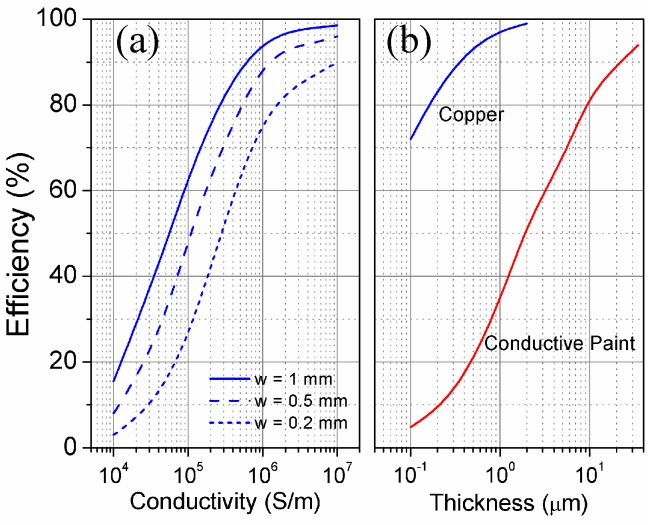
Simulated radiation efficiency of the planar tag (**a**) versus conductivity for *t* = 35 μm and; (**b**) versus conductor thickness for *w* = 1 mm. The conductive paint has a conductivity *σ* = 10^6^ S/m [10,11].

In order to quantify the effect of ohmic losses in the fully planar approach, we simulated (by means of ADS Microwave Studio commercial software) the antenna, with length *l* = 400 mm (1.22 *λ*), for different values of strip width *w*, thickness *t* and metal conductivity *σ*. A graphical representation of the simulated radiation efficiencies is depicted at Figure 5. The data reveal that it is possible to obtain high radiation efficiency even by using reduced conductivity materials, such as screen-printing conductive paint, with reasonable values of thickness (of the order of 20 μm) and conductor width (1 mm). To maintain generality, the simulations did not take into account the presence of a dielectric substrate. This could be the case of direct screen printing of the tag over the item surface. Regarding the inlay implementation, the substrate should be chosen to be as thin as possible, and to present a low permittivity, in order to preserve the electrical length of the antenna and therefore its gain. Nevertheless, the characteristics of the tagged item (not considered in this study) are relevant for the determination of the optimum antenna dimensions.

## 4. Experimental Results

In order to validate the simulated results, the tag layout described in Section 3.1 was fabricated and its read range was measured. The measurement setup consisted of a commercial Motorola FX7400 UHF-RFID reader matched to a half-wave dipole antenna, obtaining a transmission *EIRP* = 1.5 W. The tag was measured in an outdoor environment, with the ground as a unique nearby scatterer. The read range was measured in the 865–868 MHz (Europe) and 902–928 MHz (USA and others) bands available from the reader.

**Figure 6:**
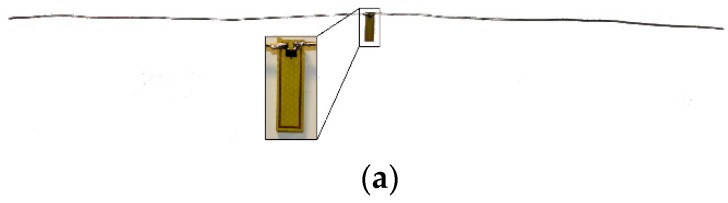

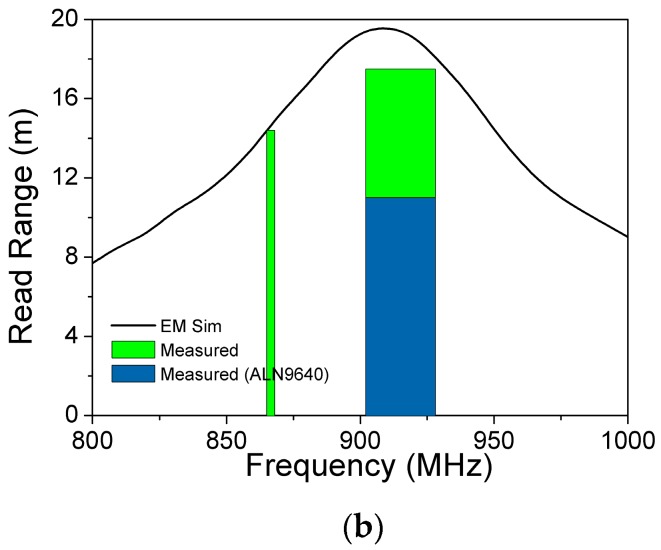
(**a**) Manufactured tag prototype; (**b**) Simulated and measured read range of the fabricated 1.25 wavelength long dipole antenna tag.

The results, normalized to *EIRP* = 4 W (which is the value used for the simulated read range, and the maximum *EIRP* value in USA [12]), and compared to the simulated read range curve, are depicted in Figure 6b. The data is in very good agreement in the 865–868 MHz band, where 14.4 m were obtained. On the other hand, the measured peak read range is 17.5 m in the 902–928 MHz band, which is 10% lower as compared to the simulated value. This discrepancy can be attributed to the fact that the actual chip sensitivity tends to be smaller [13,14] than the manufacturer nominal value. Indeed, each dB decrease in the chip sensitivity leads to 10% reduction of the read range, according to Equation (3). Nonetheless, when compared to one of the best performing available commercial tags in terms of read range, i.e., the Alien ALN9640 “Squiggle”, the tag presented in this work provides a measured read range in the 902–928 MHz band that represents an increase of almost 60% (Figure 6). This improved read range has been achieved despite the fact that the nominal sensitivity (−18 dBm [15]) of the unpackaged Higgs-3 chip used in the ALN9640 inlay is better than the one of the *SOT-323* version used in this work (−17 dBm). This improvement of read range is a remarkable aspect of this paper. It is also remarkable that the read range measured when the proposed tag is attached to a wooden pallet (1 m above the ground) is 16.4 m, pointing out the potential of these tags for such application.

## 5. Conclusions

The read range limitations of passive UHF-RFID tags based on electric dipole antennas have been studied in this work. A 1.25 wavelength long linear wire has been proposed as a tag antenna which maximizes the gain while maintaining acceptable dimensions and radiation pattern. Based on this result, a tag prototype has been designed by matching the antenna impedance to the Alien Higgs-3 chip input impedance by means of a shunt inductance. The presented tag antenna length and diameter are 400 mm and 0.8 mm, respectively, and a 4 × 14.6 mm^2^ rectangular substrate, holding the chip and the inductive matching network, is located at the center of the tag. The measured read range exhibits a maximum value of 17.5 m at the 902–928 MHz band, representing an important improvement as compared to the best general purpose commercial passive UHF-RFID tags. Moreover, several simulations predict that the tag can be implemented in planar technology (i.e., inlay) while maintaining high values of radiation efficiency. The application of this type of antennas to RFID tags is the main novel aspect of this paper.
